# Research progress on exosomal proteins as diagnostic markers of gastric cancer (review article)

**DOI:** 10.1007/s10238-022-00793-5

**Published:** 2022-01-22

**Authors:** Hang Su, Weihong Ren, Dai Zhang

**Affiliations:** 1 The First Clinical Medical Institute, Henan University of Chinese Medicine, No.156 Jinshui East Road, Zhengzhou, Henan Province China; 2 Department of Laboratory Medicine, The First Affiliated Hospital of Henan University of Chinese Medicine, No.19 Renmin Road, Zhengzhou, 450000 Henan Province China

**Keywords:** Exosomal protein, Gastric cancer marker, Diagnosis

## Abstract

Gastric cancer (GC) is one of the most common types of tumors and the most common cause of cancer mortality worldwide. The diagnosis of GC is critical to its prevention and treatment. Available tumor markers are the crucial step for GC diagnosis. Recent studies have shown that proteins in exosomes are potential diagnostic and prognostic markers for GC. Exosomes, secreted by cells, are cup-shaped with a diameter of 30–150 nm under the electron microscope. They are also surrounded by lipid bilayers and are widely found in various body fluids. Exosomes contain proteins, lipids and nucleic acid. The examination of exosomal proteins has the advantages of quickness, easy sampling, and low pain and cost, as compared with the routine inspection method of GC, which may lead to marked developments in GC diagnosis. This article summarized the exosomal proteins with a diagnostic and prognostic potential in GC, as well as exosomal proteins involved in GC progression.

## Introduction

Gastric cancer (GC) is the 5th most frequently diagnosed cancer and 3rd leading cause of cancer mortality, accounting for 1,000,000 new cases and ~ 783,000 deaths in 2018 [1]. At present, the lack of appropriate screening markers for GC leads to the majority of patients missing the best treatment opportunities. Therefore, the exploration and application of diagnostic markers for GC are of great significance. Studies have shown that exosomal proteins can play a role in the diagnosis and prognosis of GC [2, 3]. Exosomes have the potential to be diagnostic and prognostic markers of cancer, since they contain microRNA (defined as a kind of tiny non-coding RNA with 23–35 nucleotide and tumor diagnostic potential) and cell-specific proteins that can mediate cell communication [4–8]. Exosomal proteins have a broad prospect in the development and application of biomarkers in GC diagnosis and prognosis, with a low cost and less pain, as compared with conventional diagnostic methods. With further research conducted, exosomal protein markers of GC with a high specificity and sensitivity may be widely used in clinical practice. In the present study, exosomal proteins with a diagnostic and prognostic potential of GC and their research status were reviewed herein.

## Serological proteomics markers commonly used in GC diagnosis

Serological markers of GC include specific markers, such as gastrin-17 and pepsinogen (PG), and non-specific ones, such as carbohydrate antigen 199, CA724 and carcinoembryonic antigen. The changes in PG level and PGI/II ratio are considered indicators of gastric mucosa atrophy. Both PGI and PGII are known to reduce the development of atrophy and loss of specialized cells. PGI usually shows a more advanced decrease than PGII, causing a low PGI/II ratio [9]. Gastrin-17 is the main component of gastrin, which is secreted by G cells in the gastric wall. Tu et al. [10] demonstrated that a low level of gastrin-17 may be a biomarker for atrophic gastritis in the gastric antrum. Yu et al. [11] performed gastroscopy on 68 patients with chronic atrophic gastritis and 86 healthy controls, and detected serum levels of PGI, PGII and gastrin-17 by enzyme-linked immunosorbent assay (ELISA). That study subsequently reported that the sensitivity, specificity and area under curve of gastrin-17 were less than the PGI, PGII and PGI/II ratio, suggesting inferior clinical value of gastrin-17 in screening chronic atrophic gastritis than that of PG. CA724 has a high specificity and dissatisfactory clinical application [12]. Currently, the serological examination of GC uses multi-index combined detection to improve diagnostic efficiency, which remains flawed due to the inadequate sensitivity or specificity of single-detection indicators. The protective effect of exosomes makes its proteins a more favorable marker, as compared with the aforementioned serum markers.

## Brief introduction of exosomes

**Fig. 1:**
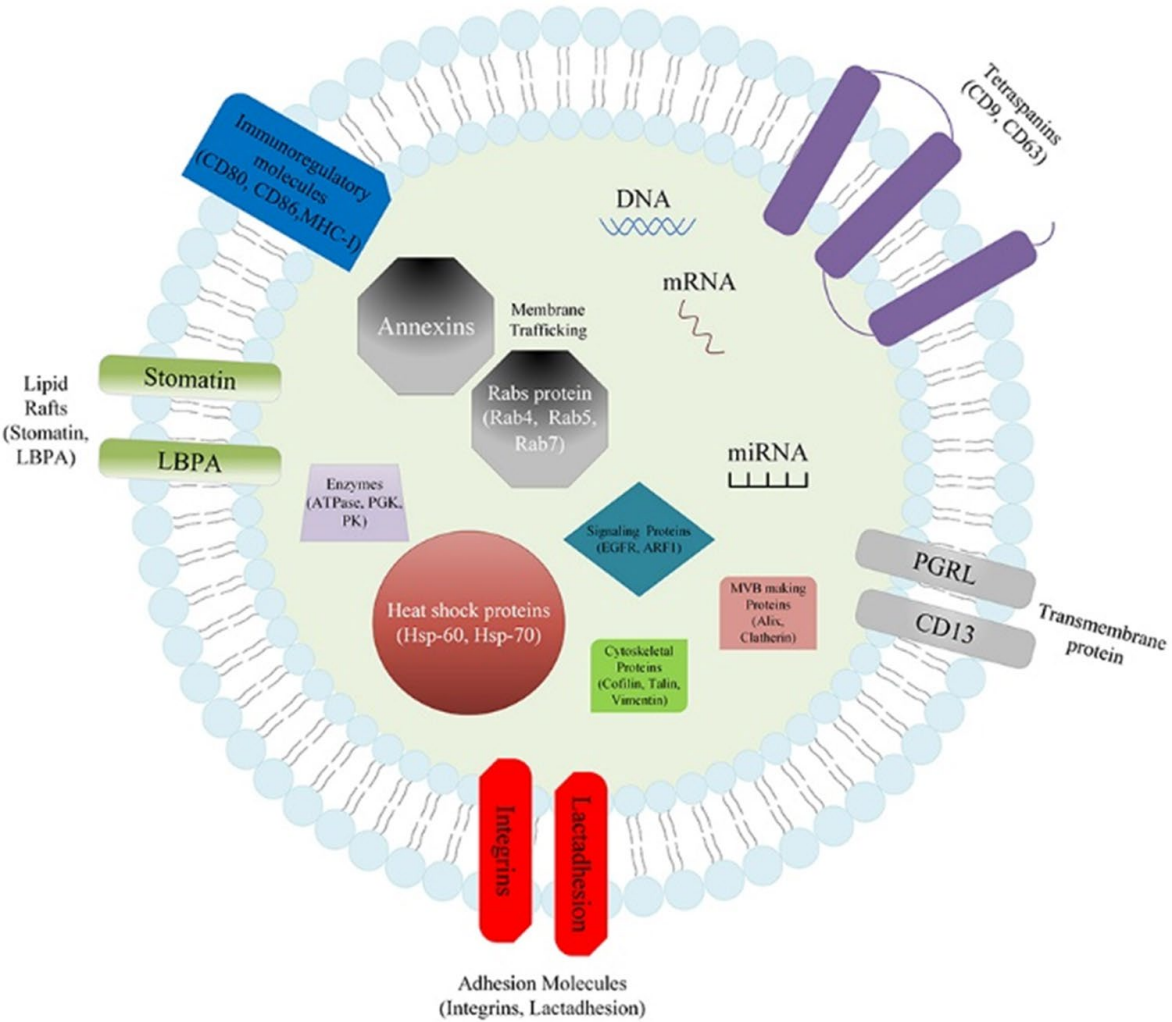
*Compositions of Exosomes*. The main components of exosomes include DNA, RNA (mRNA, miRNA) and proteins such as transmembrane protein (CD9, CD63), tetraspanins (CD13, PGRL), heat shock proteins (Hsp-60, Hsp-70), signaling proteins (EGFR, ARF1), MVB making Proteins (Alix, Clatherin), immunoregulatory molecules (CD80, CD86, MHC-I), cytoskeletal proteins (Cofilin, Talin, Vimentin), membrane trafficking (Rabs protein, Annexins), enzymes (ATPase, PGK, PK), immunoregulatory molecules (CD80, CD86,MHC-I), etc

Exosomes are a type of membranous vesicles with a diameter of 30–150 nm which contain proteins, nucleic acid, etc. (Fig. 1) [13, 14]. They can be released by a variety of cells, including fibroblasts, intestinal epithelial cells, neurons, fat cells and tumor cells [15], and exist in multiple body fluids [16, 17]. Exosomes were first discovered and named by Johnstone et al. [18]. Early studies considered exosomes the “garbage bags” inside cells. Current study has demonstrated that exosomes function in numerous biological processes and disease courses, such as angiogenesis, intercellular communication and antigen presentation [15].

### The formation of exosomes

**Fig. 2:**
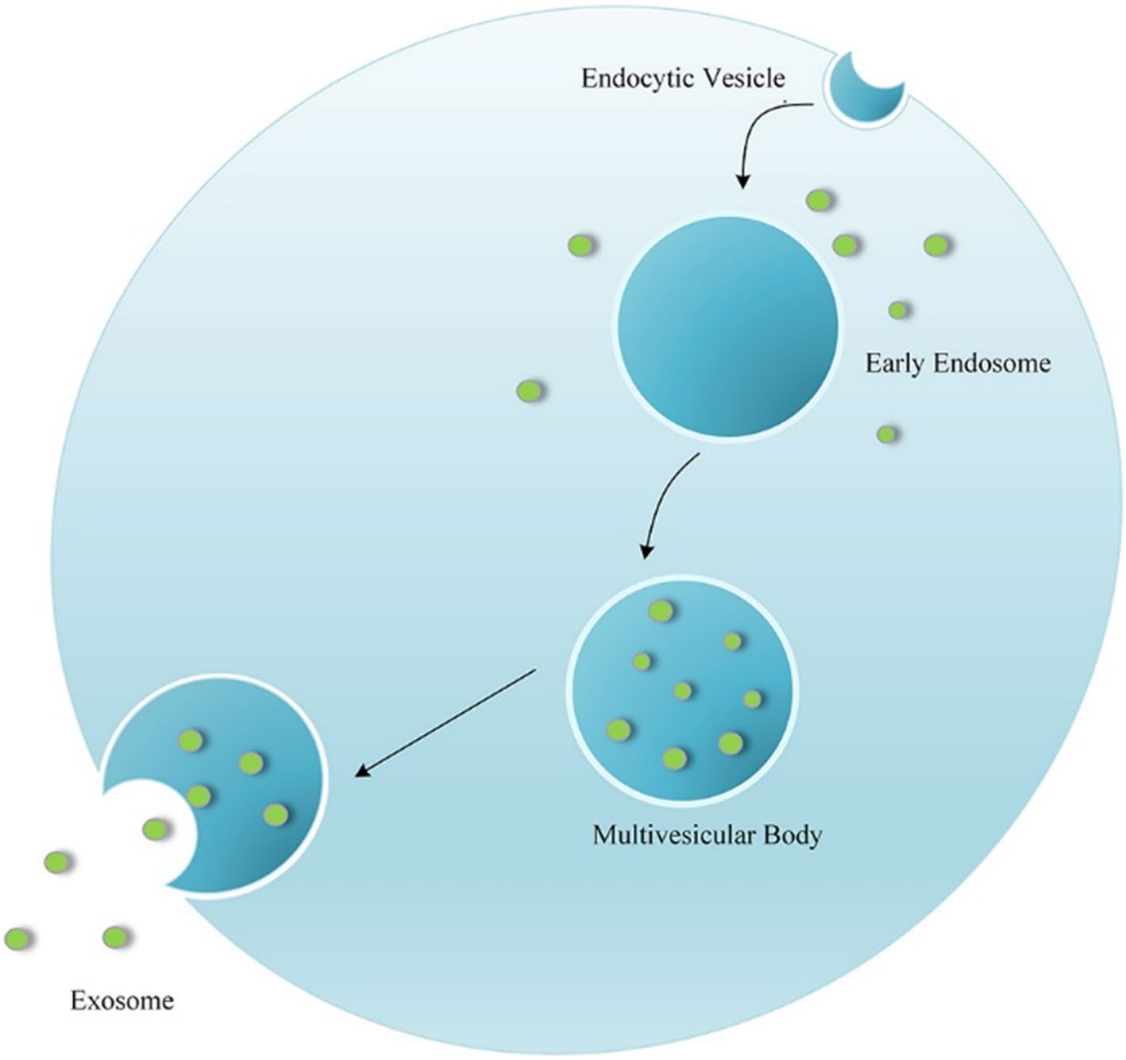
*Biogenesis of Exosomes*. The Biogenesis of exosomes contains following steps: 1. Generation of endosome. 2. Transformation of endosome into multivesicular body (MVB). 3. Exosomes release

The formation process of exosomes consists of the following three steps: First, the endosome is generated by the invagination of the plasma membrane. Secondly, the endosome is transformed into a multivesicular body (MVB). Finally, the MVB fuses with cell membranes and releases exosomes (Fig. 2) [15].

### The separation and detection methods of exosomes

The commonly used methods for exosome extraction include ultracentrifugation and related techniques, density gradients, extraction kit, size exclusion chromatography (SEC), affinity and precipitation. Several separation methods such as tangential flow, microfluidics and field flow fractionation have shown relatively few application [19]. Each of these methods possesses respective strong points and weaknesses. Centrifugation-based isolation methods, at the present stage, remain the gold standard and are most frequently applied to the exosomal separation [20, 21]. It can extract abundant exosome but is time-consuming and inefficient in recovery and repeatability, which hinders the clinical application [21–23]. Immunomagnetic beads provide better purity but can only capture exosomes with specific targeted proteins [24]. Commercial extraction kits, which are simple-handling and without costly equipment, are also incapable of avoiding chemicals contamination [24, 25]. The acquirement of highly purified exosomes is of vital importance to its researches and the extraction method still needs to be improved.

The detection of exosome involves in the diagnosis and treatment of many diseases. Conventional detection methods of exosome include optical detection methods and non-optical ones. Conventional detection techniques are mainly composed of Dynamic light scattering (DLS) and nanoparticle tracking analysis (NTA). DLS, which is able to characterize exosome with a certain steady size, can neither handle samples with different sizes nor acquire source information [21]. Compared to DLS, NTA can characterize exosomes with discrepant sizes and fluorescence, meaning it can detect distributions of antigens on exosomes [21]. Non-optical methods include transmission electron microscopy (TEM), atomic force microscopy (AFM) and enzyme-linked immunosorbent assay (ELISA). TEM combined with cryogenic technique is capable of measuring the size of exosome and is first choice of visualizing nanoparticles and proteins [26]. AFM provides the size distribution of exosomal sample. Besides, it can characterize the abundance, structure and other features of exosomes derived from tumor samples [21]. As a common detecting method in immunology, ELISA has gradually been applied to exosomal detection. In addition to these methods mentioned above, several emerging microfluidics-based detection technologies which are considered highly sensitive, low reagent-consuming and portable with high-throughput capacity have played a role in exosome characterization [21, 27].

### Exosome in delivery system

Exosome, with tiny volume, can easily pass through vascular wall and extracellular matrix and prevent phagocytosis of mononuclear macrophages [28]. Furthermore, exosome is capable of crossing biological barriers with weak immune response, making it nanocarrier and potential superior choice of delivering therapeutic drug [28–30]. With the aid of loading technology, required drugs can be transmitted to recipient cells and tissues. Pan et al. [31] created a new Exo-PMA/Au-BSA@Ce6 nanovehicle by combining urinary exosomes and ultra-small Au-BSA@Ce6 nanocomposites via an electroporation method, which improved photodynamic therapy on cancer and achieved real-time imaging of NIR fluorescence. Besides, Research by Liang et al. [32] shown that engineered exosomes delivery system which transfer miR-21i and chemo-therapeutic drug 5-FU to the HCT-1165FR cancer cells are capable of enhancing the therapeutic efficiency of colon cancer and reversing the drug tolerance. Overall, exosome-involved delivery system has the potential to make a contribution to cancer diagnosis with the development.

## Exosomal proteins with a potential for GC diagnosis and prognosis

**Fig. 3:**
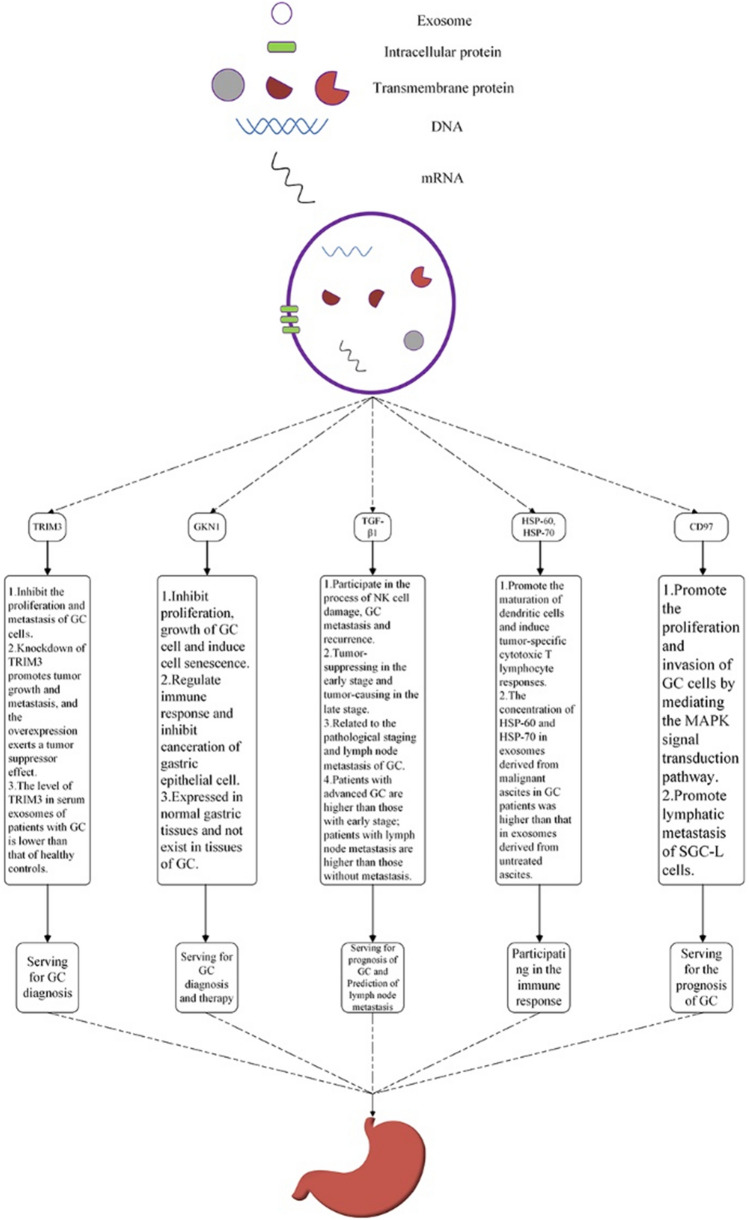
*Exosomal proteins with potential as GC marker*. TRIM3 and GKN1 can inhibit the proliferation of GC cells. CD97 has the opposite effect. TGF-β1 is able to play a dual role in GC. GKN1 and HSP-60, HSP-70 participate in the immune response. CD97, TGF-β1 could serve for GC prognosis and TRIM3, GKN1 can be applied to GC diagnosis

Exosomal proteins are divided into two types. The first type includes the common proteins in exosomes such as transmembrane transport and integration-related proteins, tetraspanins (e.g., CD9 and CD63) and heat shock proteins (HSP) [33]. The other type includes proteins present in certain types of exosomes. For example, exosomes from tumor cells possess a variety of tumor antigens. Figure 3 and Table 1 list the gene location, molecular weight and function of the proteins serving as GC markers.

**Table 1:** Potential Exosomal Protein Biomarker in GC

English abbreviations	Gene location	Molecular weight(kDa)	Source of exosomes	References
TRIM3	11p15.5	80.8	Serum, Cell Line	[2]
GKN1	2p13.3	18	Tissue	[64]
TGF-β1	19q13.1	25	Cell Line	[65]
CD97	19p13.12	75–90	Cell Line	[91]
HSP-60	2q33.1	60	Malignant Ascites	[90]
HSP-70	6p21.33	70	Malignant Ascites	[90]

### Tripartite motif 3 (TRIM3)

TRIM3, which is located at chromosome 11p15.5, is a member of the TRIM family that includes a RING domain, one or two B-boxes, and a coiled coil domain [34–38]. The upregulation of TRIM3 inhibits tumor cell behaviors, including proliferation and metastasis [2, 38–41]. Furthermore, the suppressed TRIM3 expression was associated with poor survival rates in breast cancer patients, suggesting that TRIM3 played a role in breast cancer inhibition [42]. Lu et al. [43] verified the upregulated expression of TRIM3 in Ewing sarcoma tissues, as compared with normal tissues. The enhanced expression of TRIM3 markedly and continually hindered autophagy in Ewing sarcoma (ES) cells. The opposite phenomenon was observed in TRIM3-silenced ES cells. In addition, TRIM3 is a potential target of miRNA (miR)-454-3p to promote the proliferation of human cervical cancer cells and inhibit their apoptosis [44]. With regards to other diseases, the experiment results by Wang et al. [45] indicated that TRIM3 was more lowly expressed in rheumatoid arthritis synovial tissues than healthy controls. Moreover, the stimulation of lipopolysaccharides on cell multiplication, the release of tumor necrosis factor-α, interleukin-6 (IL-6) and IL-1β, as well as the therapeutic effect on collagen-induced arthritis in rats were suppressed by the increased expression of TRIM3. Another study has also supported the viewpoint that TRIM3 contributes to the early diagnosis and treatment of Parkinson’s disease [46].

Exosomal TRIM3 in patients’ serum has the potential to serve as a diagnostic marker for GC. Fu et al. [2] extracted exosomes from the serum of GC patients and cell culture supernatant using an exosome extraction kit, and tested the proteomic characteristics of exosomes by liquid chromatography-tandem mass spectrometry (LC–MS/MS). The expression of TRIM3 in the serum and tissue was also detected by ELISA and western blotting (WB). The results reported that expression levels of TRIM3 protein in both serum and tissue were lower than those in healthy controls. The overexpression of TRIM3 facilitated the growth and metastasis of GC which contradicted with TRIM3 knockdown. All these results showed that TRIM3 has the potential to serve as a diagnostic biomarker for GC.

### Gastrokine-1 (GKN1)

GKN1, also called antral mucosal protein (AMP)-18, is a member of the GKN family that is located at chromosome 2p13.3 [47]. It is a stomach-specific protein of 181‑184 amino acids with a molecular weight of 18 kDa [48–52]. GKN1 comprises a hydrophobic signal peptide in the extreme NH2-terminal, and a BRICHOS domain containing conserved amino acid residues, aspartic acid residue, cysteine acid residues and a COOH‑terminal domain [52–58]. GKN1 is secreted by gastric epithelial cells, stored in cytoplasmic granules and released into the extracellular environment through exosomes [49–51]. It is expressed in normal gastric tissue but not in GC tissue [59]. Studies have confirmed that GKN1 impacts cellular behavior. For example, it can hinder cell proliferation by inhibiting the expression of gastrin receptor [60]. In addition, GKN1 restrains cell growth and induces cell senescence by activating the p16/Rb and p21 signaling pathways [61]. It can also regulate immune response and suppress gastric epithelial cell carcinoma by affecting the expression of ILs and nuclear factor kappa B (NF-κB) [62]. GKN1 has been proven to be involved in the diagnosis and progression of various types of GC. Moss et al. [54] reported that a suppressed GKN1 expression was regularly observed in gastric adenocarcinomas, particularly of the diffuse subtype. The study by Dokhaee et al. [47] confirmed that the GKN1 mRNA in the gastric tissue of GC patients was significantly lower than that in normal gastric tissue. Yoon et al. [63] measured the serum GKN1 of 200 healthy individuals and 1268 cancer patients (including 500 patients with GC) and analyzed its clinical value. They found the serum concentration of GKN1 of healthy individuals was much higher than that of patients with GC. The diagnostic accuracy of the serum GKN1 protein at the optimum cutoff was 0.9675. Moreover, GKN1 in serum of patients with advanced gastric cancer (AGC) were lower than those of patients with early gastric cancer (EGC). The diagnostic accuracies of GKN1 at the optimum cut-off (0.9675) were 0.8912 and 0.9589 for EGC and AGC, respectively. All these evidences have shown the specificity and prospect of serum GKN1 as a biomarker for AGC and EGC. In addition, another study indicated that serum GKN1 concentrations discriminated healthy individuals with normal stomach and subjects with atrophy without and with intestinal metaplasia (IM) from GC patients with AUCs of 1.0000, 1.0000, and 0.9964, respectively. Also, serum GKN1 levels in patients with GC yielded AUCs of 0.9938 and 0.9987 distinguishing patients with hepatocellular and colorectal carcinomas from GC patients. Besides, serum GKN1 concentrations distinguished patients with EGC from normal individuals and subjects with atrophic gastritis without and with IM from GC patients with AUCs of 1.0000, 1.0000 and 0.9892, respectively. All of these data shown the diagnostic value of serum GKN1 on GC [50].

The diagnostic potential of exosomal GKN1 has been illustrated by previous studies. Yoon et al. [50] found that the GKN1 protein was expressed in exosomes derived from gastric epithelial HFE-145 cells but not in exosomes from AGS and MKN1 cells. Exosomes containing GKN1 impeded the proliferation and induced the apoptosis of AGS and MKN1 cells. In addition, GKN1-positive exosomes could inhibit tumor growth in GC mice model, indicating the potential of exosomal GKN1 in the diagnosis of GC. However, this conclusion has not been validated in a large-scale sample and evaluated in a prospective study. Two years after that study, Yoon et al. [64] conducted an investigation (containing 5 sporadic GC patients who had undergone a gastrectomy) on the specific uptake of exosomes from normal human gastric mucosa epithelial cells HFE-145 and examined the anticancer activity of exosomal GKN1 by immunofluorescence, WB and protein microarray chip. Experimental results showed that the exosomal GKN1 inhibited GC growth by binding to HRas and inhibiting its combination with b-raf and c-raf, which then decreased HRas/Raf/MEK/ERK signaling in AGS, MKN1 and xenograft tumor tissues. Moreover, exosomal GKN1 was involved in the inhibition on the migration and invasion of GC cells by suppressing epithelial-mesenchymal transition (EMT), suggesting that GKN1 can also be used for GC treatment.

### Transforming growth factor-beta 1 (TGF-β1)

TGF-β1, whose gene is located on chromosome 19q13, is a 25-kDa homodimeric polypeptide secreted by immune and tumor cells [65–71]. TGF-β1 signaling is involved in the regulation of many biological processes, including cell proliferation, differentiation, migration and apoptosis [72, 73]. Several studies have proven that TGF-β1 plays a dual role in the tumor development process. In the early stage of tumors, TGF-β1 plays a suppressive role by hindering cell proliferation and inducing apoptosis [74–76]. Nevertheless, it was classified as a carcinogen, as it participates in EMT and angiogenesis in the late phase of tumors [77]. In terms of GC, TGF-β1 has the potential to be a predictor of GC progression and is also involved in natural killer (NK) cell injury, metastasis and recurrence of GC. A study showed that the TGF-β1 level was increased in patients with GC, and this increase was positively correlated with tumor stage [78]. The potential of plasma TGF-β1 as a predictor of GC progression was demonstrated by Zou et al. [79]. Furthermore, TGF-β1 secreted by cancer-associated monocytes/macrophages induced functional injury in NK cells in GC [80]. The study by Han et al. [81] identified a negative correlation between the TGF-β1 concentration and NK cells that expressed NKp30, NKp46, NKG2D and dnam-1 in peripheral blood. They also further illustrated the immunosuppressive effect of TGF-β1 on NK cells in patients with GC. Coban et al. [82] demonstrated that mean serum TGF-β1 levels were higher in patients with gastric cancer (GC) or colon cancer compared to the control group (p = 0.001). The sensitivity of TGF-β1 was better in patients with GC than in patients with colon cancer. TGF-β1 had higher sensitivity than carcinoembryonic antigen (CEA) in GC patients. In addition, TGF-β1 was found to participate in GC cell EMT induction, invasion and migration [83, 84]. Wang et al. [85] suggested that TGF-β1 was specifically involved in the postoperative distant recurrence of gastric adenocarcinoma. The TGF-β1 signal was capable of upregulating the expression of vascular endothelial growth factor-c (VEGF-C) which caused lymphangiogenesis in GC [77].

The correlation between TGF-β1 and the prognosis of gastric adenocarcinoma patients has been confirmed. Yen et al. [65] analyzed the expression of exosomal TGF-β1 from gastroepiploic veins of 61 gastric adenocarcinoma patients by ELISA. Their findings were as follows: The expression of exosomal TGF-β1 in stages II, III and IV GC patients was higher than that in stage I patients. In addition, the expression of exosomal TGF-β1 in patients with lymph node metastasis (LNM) was twice as high as that in patients without LNM, suggesting that the expression of exosomal TGF-β1 in patients with gastric adenocarcinoma was associated with pathological stage and LNM. Moreover, exosomal TGF-β1 may be of greater value in the prediction of GC LNM than serum TGF-β1 because of the controversial association between serum TGF-β1 levels and the clinicopathological characteristics of gastric adenocarcinoma [86–88].

### Other proteins

In addition to the aforementioned ones, some proteins in both exosomes and other body fluid including serum and bile are associated with the proliferation, invasion and metastasis of GC. HSPs exist extensively and are highly conservative. Hoshino et al. [89] demonstrated that HSP-70 had the highest specificity and 3rd sensitivity among six tumor-associated antigens (p53, heat shock protein 70, HCC-22–5, peroxiredoxin VI, KM-HN-1, and p90) and their panel. A study by Zhong et al. [90], including 18 gastric adenocarcinoma patients, showed that the concentrations of HSP-60 and HSP-70 in exosomes from heat-treated malignant ascites in gastric adenocarcinoma patients were higher than that from untreated ones. In addition, exosomes from heat-treated malignant ascites have been shown to contribute to the maturation of dendritic cells and cause a tumor-specific cytotoxic T lymphocyte response, suggesting an association between HSP and gastric adenocarcinoma. CD97 is a member of EGF-seven transmembrane (EGF-TM7) with a molecular weight of 75–90 kDa. Li et al. [91] found that CD97 facilitates the proliferation and invasion of GC cells through the exosome-mediated mitogen-activated protein kinase (MAPK) signaling pathway by exosome extraction proliferation and Matrigel invasion assays. Liu et al. [92] isolated exosomes from SGC-L cells (a cell line derived from SGC-7901 with a high lymphatic metastasis) and SGC-L cells with CD97-knockdown (SGC-L/CD97-kd), and co-cultured them with GC cells. The results revealed that exosomes from SGC-L cells increased proliferation and invasion by 20 and 30%, respectively. Also, intrafootpad injections of SGC-L cells can significantly conduce to the two aforementioned cell lines accumulation in the draining lymph nodes and enhance the expression of CD55, CD31, CD151, CD44v6, α5β1 and epithelial cell adhesion molecule, which suggested that the exosome-dependent CD97 plays a pivotal role in lymphatic metastasis in SGC-L cells.

## Exosomal proteins involved in GC progression

### GKN1 and TRIM3

In addition to their diagnostic potential, exosomal GKN1 and TRIM3 also get involved in the progression of GC. Exosomal GKN1 could be internalized by the gastric epithelium and delay the multiplication of AGS and MKN1 cells as previously mentioned, which could be a significant mechanism of body's self-protection to cope with GC [50]. A study by Fu et al. [2] showed that the growth and metastasis of GC can be restrained by the regulation of stem cell factors and EMT regulators induced by exosomal TRIM3.

### TGF-β superfamily

The TGF-β superfamily contains a huge range of cytokines participating in different biological activities, such as polypeptide function in the regulation of tumor cells, tumor-related fibroblasts and immune-related cells that exist in the tumor microenvironment [93–95]. The TGF-β superfamily includes TGF-β, activin and bone morphogenetic protein (BMP), which play a crucial role in regulating the growth, adhesion, migration, differentiation and apoptosis of cells. There are three subtypes of TGF-β found in mammals: TGF-β1, TGF-β2 and TGF-β3 [96]. TGF-β1, most generally expressed in human tissues, has three receptors: transforming growth factor beta receptors 1 (TGF-βR1), TGF-βR2 and TGF-βR3 [97]. A strong correlation between TGF-β and GC has been confirmed. A study by Liu et al. [93] indicated that the decrease in the mRNA expression of TGF-β1 and its receptors was associated with the improved overall survival (OS) of GC patients. In addition, their knockout restrained the migration, invasion and proliferation of gastric carcinoma cells. Yang et al. [97] found that advanced GC patients with a low TGF-β1 expression often had a favorable OS, while the high levels of TGF-β1 were markedly associated with poor OS, and GC patients with a high TGF-β2 expression had a better OS than that of patients with a low TGF-β2 expression, as determined by Cancer Genome Atlas database analysis. BMPs, which are members of the TGF-β superfamily, are glycoproteins produced by certain cells. BMPs could fall under different subfamilies, based on amino acid residues sequence homology: (i) BMP-2 and BMP-2B (BMP-4); (ii) BMP-3 and BMP-3B; (iii) BMP-5, BMP-6, BMP-7, BMP-8, BMP-9, BMP-10, and BMP-11; (iv) BMP-12, BMP-13, BMP-14 and BMP-15. However, BMP-1 does not belong to this family [98]. BMPs participate in two signaling pathways: The Smad-dependent and Smad-independent pathways [98, 99]. Studies have suggested that BMPs are involved in the progression of GC. Deng et al. [100] demonstrated that BMP4 was overexpressed in GC cell lines and facilitated the EMT and metastasis of GC cells in vitro and in vivo, the knockdown of which markedly suppressed the EMT and metastasis of GC cells. Moreover, the expression of BMP4 was constantly upregulated in gastric carcinoma tissues and involved in the harsh prognosis of GC patients [100]. Lei et al. [101] performed a study on BMP10 in GC cell lines, 52 GC tissues and normal tissues. That study confirmed that the high BMP10 expression in AGS and SGC-7901 cell lines hindered the growth and migration of these cells, with BMP10-knockdown promoting their growth, migration, and metastasis. They also found that BMP10 was downregulated in GC tissues. These results showed the inhibitory effect of BMP10 on GC. BMP1, as the special one, was also found to be associated with GC. A study by Hsieh et al. [102] suggested that the inhibition of BMP1 impeded GC cell migration, which had a negative impact on metastasis. In addition, BMP1-overexpression was linked with a very poor long-term survival of GC patients at an advanced stage.

Exosomal TGF-β1 and BMP have also been found to participate in the progression of stomach cancer. In addition to predicting the LNM of GC, exosomal TGF-β1 is also involved in the advancement of GC. It can assist GC cells in escaping immunological surveillance by causing regulatory T cell differentiation, making GC hard to manage. Furthermore, exosomal TGF-β from SGC-7901 cells has been proven to lead to the activation of the Smad pathway, and the succedent differentiation of human umbilical cord mesenchymal stem cells (hucMSCs) into cancer-associated fibroblasts (CAFs) through interacting with TGF-βR1 in hucMSCs [103]. Ning et al. [104] extracted exosomes from human gastric mucosal epithelial cells GES-1 and GC cells SGC-7901 and tested CAFs marker expression and CAFs conversion-related signaling pathways through WB, immunofluorescent staining and reverse transcription quantitative polymerase chain reaction (RT-qPCR). It was revealed that BMP2 in exosomes activated the PI3K/AKT and MEK/ERK signaling pathways and resulted in the transition of pericytes to CAFs.

### Epidermal growth factor receptor (EGFR)

EGFR, also known as human epidermal growth factor receptor 1 (HER1), is a member of the HER (ErbB) family. Other members of this family are ErbB-2 (HER2), ErbB-3 (HER3), and ErbB-4 (HER4) [105]. EGFR, and the rest of the ErbB family members, contain an extracellular domain, lipophilic transmembrane region, intracellular domain containing tyrosine kinase and carboxy-terminal region [106]. EGFR is transmembrane glycoprotein, which belongs to the receptor tyrosine kinase group and contains an extracellular domain, lipophilic transmembrane region, intracellular domain ontaining tyrosine kinase and a carboxy-terminal region, like the rest of the ErbB family members [106, 107]. The activation of EGFR has been shown to improve cell growth, proliferation and differentiation, as well as wound healing [108, 109]. Ample evidence has revealed a strong association between EGFR and gastric diseases. The phosphorylation of EGFR has been linked to DNA injury in gastric epithelial cells infected with Helicobacter pylori (H. pylori). Enhanced levels of activated EGFR in the epithelium were observed in gastritis and atrophic gastritis, but were not observed in intestinal metaplasia, and intestinal and diffuse type gastric carcinoma. In addition, the phosphorylated EGFR level in the gastric tissues of patients with gastric disease developing to intestinal metaplasia or dysplasia were higher than those of patients without disease progression [110]. The overexpression of EGFR, which is associated with poor prognosis, was found in 37% of gastric/esophageal adenocarcinomas [111, 112]. Zhang et al. [113] suggested that overexpressed EGFR notably heralded an adverse outcome, and could function as an indicator of poor prognosis in GC patients. In a study by Terragni et al. [114], 8 cases with a strong EGFR expression were identified among 19 canine cases (5 gastric adenomas, 5 intestinal type gastric carcinomas and 9 diffuse-type gastric carcinomas) despite the position or biological behavior of the tumor.

Exosomal EGFR was found to be linked to GC progression. Zhang et al. [115] isolated exosomes from cell culture medium and the serum of gastric adenocarcinoma patients. Exosomes extracted from SGC7901 were co-cultured with primary mouse liver cells. Next, the effect of these exosomes on hepatocyte growth factor (HGF) in liver cells was examined, and the liver HGF’s impact on promoting the invasion and metastasis of SGC7901 cells in vitro, as well as its effects on the formation and growth of liver metastases in vivo, were evaluated. That study demonstrated that EGFR was enriched in exosomes derived from patients with gastric adenocarcinoma, an effect opposite to that observed in exosomes of the healthy control. EGFR could be transported to the liver and integrated on the plasma membrane of liver stromal cells. Exosomal EGFR suppressed the expression of miR-26a/b and activated HGF, which combined with the cell mesenchymal-epithelial transition factor (c-MET) receptor on the migrated cancer cells and promoted the landing and proliferation of these cells, offering beneficial conditions for the hepatic metastasis of GC.

### Cytotoxin-associated gene A (CagA)

CagA is a protein encoded by CagA with a molecular weight of 128–145 kDa. It contains a structured N-terminal region that includes domain I, domain II and domain III and accounts for 70% of the entire CagA, and an intrinsically disordered/unstructured C-terminal tail, whose polymorphisms in structure induce the change of CagA molecular weight [116–118]. CagA, on the basis of a repetitive amino acid sequence known as glutamine acid-proline-isoleucine-tyrosine-alanine at its 3’ terminal, can be segmented into two types, namely CagA of Western Asia and that of East Asia [119]. CagA-positive *H. pylori* is linked to gastric adenocarcinoma and gastric mucosa-associated lymphoid tissue lymphoma of B cell origin, and CagA serves as a main virulence factor of *H. pylori*, which makes *H. pylori* cagA-positive strains the most severe risk factor of GC. The transmission of CagA into the cytoplasm of the host cell has been shown to play a vital role in H. pylori pathogenesis and GC progression [120, 121]. Na et al. [122] reported that patients with poorly differentiated adenocarcinoma and signet ring cell carcinoma of individuals with early GC (EGC) and H. pylori CagA-positive infection exhibited an enhanced methylation of Runt-related transcription factor 3. Simán et al. [123] also drew the conclusion that *H. pylori* CagA-positive infection is a risk factor of non-cardia gastric adenocarcinoma. Takahashi-Kanemitsu et al. [124] revealed that the non-physiological scaffolding actions of CagA granted cells numerous phenotypic tumor markers and intensified the malignant conversion, namely continuing proliferation, invasion, growth suppressors elusion, etc. In addition, under chronic inflammation, the tumorigenic activity of CagA was further reinforced [125]. Palrasu et al. [126] suggested that CagA caused the phosphorylation of X-linked inhibitor of apoptosis protein E3 ubiquitin ligase and intensified ubiquitination and proteasomal degradation of the host proapoptotic factor Siva1 which was associated with the restricting effects of *H. pylori* on apoptotic responses. Furthermore, that study proved that CagA hampered apoptosis and caused an effect that may enhance the survival of impaired epithelial cells and contribute to gastric tumorigenesis.

CagA in exosomes has also been linked to the development of gastric disease. Asako Shimoda et al. [127] detected exosomal CagA in the serum of 4 GC patients with CagA-positive *H. pylori* infection. They found CagA-containing exosomes caused morphological changes in GC and gastric epithelial cells, which suggested that functional CagA transported by exosomes into cells could be associated with the progression of extragastric disorders involved in CagA-positive *H. pylori* infection.

### Apolipoprotein E (ApoE)

ApoE is a small secretory glycoprotein with a molecular weight of 39 kDa located at chromosome 19q13.2 [128, 129]. The genetic structure of apoE is polymorphous with two single nucleotide polymorphisms in the coding region, which are divided into three different alleles (*ε*2, *ε*3 and *ε*4) and six apoE genotypes [three homozygotes (*ε*4/*ε*4, *ε*3/*ε*3 and *ε*2/*ε*2, and three heterozygotes (*ε*4/*ε*3, *ε*3/*ε*2, *ε*4/*ε*2)] [128, 129]. ApoE combines with the low-density lipoprotein receptor and thus impacts cholesterol transport, lipid metabolism and protein synthesis; Moreover, it is involved with various signal transductions by bonding with the receptors on lipid particles [130–133]. Evidence has verified the participation of ApoE in various biological processes, including antioxidant activity, tissue repair, immune response and regulation, and cell growth, proliferation, differentiation and metastasis [134–139]. In terms of gastric carcinoma, studies have proven that ApoE influences GC progression and prognosis. Sakashita et al. [140] detected the ApoE mRNA through RT-qPCR and revealed that its expression in GC tissues was enhanced, as compared with that in normal mucosas. ApoE mRNA was overexpressed with further invasion into the muscle and serosa, and more positive LNM. Moreover, GC patients with ApoE-overexpression had a worse survival than those with ApoE-downregulation. These findings suggested that ApoE might be a biomarker of survival and predicting gastric invasion. Kang et al. [141] conducted a study on 550 GC patients and an equal number of healthy individuals and observed that the existence of APOE ε2 and reduction of total cholesterol were associated with an increased risk of GC. Furthermore, there was a connection between APOE *ε*2 and an increased risk of intestinal and diffuse histotypes of GC; completely different conditions were obtained with regards to the association between APOE *ε*2 and tumor node classification or stage of GC patients, with APOE polymorphic alleles found to be linked to a developmental risk of GC and irrelevant to its progression.

A correlation between exosomal ApoE and GC progression was verified. Research of Zheng et al. [142] indicated that ApoE was recognized as a protein with a sharp specificity in exosomes from M2 macrophages. In addition, functional ApoE exosomes delivered from tumor-associated macrophages (TAMs) to cancer cells could induce the activation of the PI3K/Akt signaling pathway, increasing the metastasis of GC cells.

### Ubiquitin protein ligase E3 component n-recognin 2 (UBR2)

UBR2 is a member of the E3 family with a molecular weight of 200 kDa [143, 144]. It is associated with chromatin and spermatogenesis, and regulates chromatin dynamics and gene expression in germ and somatic cells [144, 145]. Kwon et al. [146] revealed that the UBR2 gene was overexpressed in germ cells and involved in the susceptibility to infertility caused by the meiotic arrest and apoptosis of germ cells. As an ingredient of the N-end rule pathway, UBR2 induces ubiquitination and degradation [147]. It has been proven to serve as an ubiquitin ligase in regulating the activation of nucleotide-binding domain leucine-rich repeat pyrin domain-containing 1B inflammasome caused by anthrax lethal toxin via working with the ubiquitin-conjugating enzyme UBE2O [148]. In addition, UBR2 is likely to be an indispensable part on regulating the muscle protein degradation in tumor cachexia [149]. Besides, UBR2 is associated with tumors. It is currently known as the only N-end rule pathway E3 whose expression is increased by cachectic stimuli, including tumor and proinflammatory cytokines [147].

Despite the small amount of studies on exosomes, UBR2 and GC, exosomal UBR2 remains linked to stomach cancer. A previous study by Mao et al. [150] found that UBR2 existed greatly in exosomes stemming from mesenchymal stem cells. Next, that study confirmed an abundance of UBR2 in exosomes produced by p53-deficient mouse bone marrow mesenchymal stem cells (p53^−/−^mBMMSC), as compared with p53 wild-type mouse bone marrow mesenchymal stem cells (p53^+/+^mBMMSC). In addition, exosomes rich in UBR2 from p53^−/−^mBMMSC were delivered into p53^+/+^mBMMSC and murine foregastric carcinoma cells and then enhanced UBR2 expression in these cells, amplifying growth and migration through the activation of the Wnt/β-catenin pathway.

### High mobility group box 1 (HMGB1)

HMGB1 is a member of HMGB which belongs to the HMG protein superfamily. HMG is considered as the 2nd most plentiful protein in cells [151]. It can enhance the fine tuning of transcription for dealing with fast environmental changes by interacting with nucleosomes, transcription factors, nucleosome remodeling complexes, and histone H1, and has a global role in establishing chromatin domains [151, 152]. The HMGB family is a family of chromosomal proteins participating in DNA replication, recombination, transcription and repair [153–155]. Previous studies have demonstrated that HMGB is associated with some tumors and their characteristics, such as tumor proliferation, metastasis, apoptosis escape and invasion of tissue [156, 157]. HMGB1, extensively expressed in the nucleated cells of mammals, is a nuclear and extracellular protein with a molecular weight of 25–30 kDa which combines with nuclear proteins [158, 159]; it has extensive biological functions. First, HMGB1 is involved in the aforementioned DNA-related activities [160]. Secondly, it can be secreted into the extracellular space via active secretion or passive release and combine with the corresponding receptors and then activate several pivotal cell-signaling pathways, thus participating in the initiation and progression of tumors and other diseases [161–163]. In terms of GC progression, Zhang et al. [164] found that HMGB1-overexpression was linked to the migration of gastric adenocarcinoma (GAC), and its knockdown hindered the growth and invasion of GAC cells via the NF-κB pathway in vitro and in vivo. A study by Wang et al. [165] indicated that HMGB1 partially participated in the promotion of GC cell proliferation and the potential of metastasis induced by intensive human antigen R. A study by Tian et al. [159] reported the suppression of stomach cancer cell proliferation and colony formation, and the induction of apoptosis following HMGB1-knockdown. Furthermore, its silencing markedly impeded the migration and invasion of GC cells. Moreover, HMGB1 is linked to GC angiogenesis and apoptosis. Chung et al. [166] conducted a series of analyses on 27 GC patients (10 EGC, 10 advanced GC-M0 and 7 advanced GC-M1), as well as GC cells, and reported that the enhanced expression of HMGB1 promoted angiogenesis through the mediation of IL-8. In addition, the joint targeting of HMGB1 and IL-8 was able to regulate GC angiogenesis [166]. Tao et al. [167] found that aloin reduced the expression and release of HMGB1 and then prevented the recombinant human HMGB1-induced Akt-mTOR-P70S6K and ERK-P90RSK-cAMP regulatory element binding signaling pathways from activating, which caused apoptosis in GC HGC-27 cells. There were also correlations between HMGB1 and the diagnosis and prognosis of GC. Ghweil et al. [168] showed that HMGB1 had a relatively good diagnostic efficiency in differentiating patients with gastritis from those with GC (diffuse and intestinal GC). An increased level of serum amyloid A and HMGB1 signified a higher grade and advanced stage of GC [167]. As compared with normal tissues, the expression of HMGB1, HMGB2 and HMGB3 was promoted in GC tissues, which could portend a bad prognosis of GC patients [151].

HMGB1 in exosomes has been proven to be associated with GC metastasis. Zhang et al. [169] conducted an analysis of exosomal proteins derived from GC cells (BGC-823, MGC-803 and SGC-7901) via WB and LC–MS/MS. It was then found that HMGB1 played a crucial role in the impetus of the pro-tumor activation of neutrophils. In addition, GC exosomes were found to transport HMGB1 to interact with toll-like receptor 4, which induced the activation of the NF-κB pathway, an enhanced autophagic response and promotion of GC cell immigration. Previous study also revealed that extracellular vesicles (EVs) of GC cell were capable of causing pro-tumor activation of neutrophils, which had as similar effect to that of HMGB1 mentioned above [170]. Their study also demonstrated that EVs, including exosomes, stemmed from the microenvironment of GC transported HMGB1 to induce the activation of signal transducer and activator of transcription 3 (STAT3) and the high expression of the programmed death-ligand 1 (PD-L1) gene, which could be reversed via STAT3 pathway prohibition and HMGB1 silencing. These findings offered an updated insight on neutrophils’ pro-tumor effect in GC and revealed multiple functions of EVs in coordinating an immunosuppressed microenvironment.

### MET

MET, also known as the c-MET or HGF receptor, is a transmembrane protein located on chromosome 7 [171, 172]. The extracellular domain of mature MET mainly includes a semaphorin (sema) domain (25–516), a plexin-sema-integrin domain (517–562), and 4 immunoglobulin-like regions in the plexins and transcription factors domain (563–932). Its intracellular region consists of a juxtamembrane sequence, a catalytic region and a carboxy-terminal multifunctional docking site [173]. As an HGF receptor, MET is involved in the activation of HGF, which is normally regulated by mechanisms such as paracrine ligand delivery. Even with these mechanisms, MET-related signaling pathways are also associated with oncogenesis and the progression of several types of cancer, including GC [174, 175]. MET can also impact the progression of cancer by activating other critical pathways (e.g., PI3K/AKT, Ras/MAPK, etc.) [176–178]. Furthermore, there is a correlation between MET and the bleak prognosis of GC patients. Ha et al. [179] reported that GC patients with a highly expressed MET had worse OS and disease-free survival than patients without MET overexpression. A study by Drebber et al. [180] revealed that the immunoreactivity of MET and p21 were independent indicators of prognosis in gastric adenocarcinoma. A study conducted by Zhang et al. [181] confirmed the intensified expression of MET in gastric adenocarcinoma patients with a poor prognosis. In addition, the enhanced expression rate of MET was higher in gastric adenocarcinoma patients with a positive HER2, suggesting that MET could serve as a predictive biomarker for GC patients.

Exosomal MET also participates in tumor progression. Che et al. [78] investigated the communication exosomes involved between GC cells infected by H. pylorI and macrophages. They found that the MET expression, particularly the phosphorylated active form in exosomes secreted by H. pylori-infected GC cells, was enhanced. The exosomes involving MET were transferred to macrophages and then internalized, after which exosomal MET seemingly induced the conversion of macrophages to a pro-tumorigenesis phenotype. All these discoveries confirmed that the expression of activated exosomal MET was intensified by *H. pylori* infection, and exosomal MET could have a pro-tumorigenic effect on macrophages.

## Challenges and prospects

In spite of the advantage of exosome on diagnosis and prognosis of GC, obstacles to the application of exosomal proteins should not be ignored. First, the lack of standardized extraction methods of exosomes makes it difficult to eliminate heteroprotein contamination, which has a negative impact on the research of exosomal proteins. Secondly, despite the proven potential of GC markers and their reported excellent stability and sensitivity, potential exosomal proteins markers mentioned above, such as TRIM3, lack deep fundamental research on specificity, sensitivity and application in GC diagnosis and prognosis, which is unconducive to their clinical application. Research on exosome proteomics is a fantastic way of exploring GC marker. Ding et al. [182] discovered 443 exosomal proteins, among which 110 proteins were differentially expressed, by quantitative proteomics analyses. Besides, 10 highly vital proteins (UBA52, PSMA1, PSMA5, PSMB6, PSMA7, PSMA4, PSMA3, PSMB1, PSMA6, and FGA) were observed from 110 differentially expressed proteins. However, researches on exosome proteomics for diagnosis are highly limited, which should raise concern, especially when it comes to early diagnosis that is of primary importance in GC diagnosis. Thirdly, the large demand of exosomal samples and complex process of exosomal protein analysis are due to the absence of a general amplification technique [183]. The development of rapid and high throughput detection of exosomal proteins is of great significance to their clinical application. On the other hand, exosomal proteins have shown several diagnostic advantages compared to conventional GC markers. More researches on exosomal proteins are still definitely needed. Among all these barriers, extraction and purification of exosomes, which are the basis for subsequent research, need to be improved urgently.

## Conclusion

Exosomes have been proven to participate in various biological processes, such as angiogenesis, antigen presentation, apoptosis and intercellular signaling [15]. Exosomal proteins can also be involved in the progression of many diseases, including cancer. Proteins with a potential diagnostic and prognostic role in GC, as confirmed by previous studies, are listed in Table 1. Exosomal proteins have been reported to have a preferable stability and sensitivity, and provide relevant information “in real time” during tumorigenesis and tumor development, as compared with conventional diagnostic GC markers [115]. Moreover, inspiring diagnostic performance is expected to appear for the advantage of quick sampling, less pain and low cost of exosomal protein detection. Nevertheless, challenges such as the lack of appropriate extraction methods and intensive study on exosomal proteins remain obvious. In conclusion, exosomal proteins are capable of overcoming the aforementioned obstacles and being used in the diagnosis and prognosis of GC as relevant research progresses.
